# Do different robot appearances change emotion recognition in children with ASD?

**DOI:** 10.3389/fnbot.2023.1044491

**Published:** 2023-03-02

**Authors:** Maria J. Pinto-Bernal, Sergio D. Sierra M., Marcela Munera, Diego Casas, Adriana Villa-Moreno, Anselmo Frizera-Neto, Martin F. Stoelen, Tony Belpaeme, Carlos A. Cifuentes

**Affiliations:** ^1^IDLab, Ghent University—imec, Ghent, Belgium; ^2^Bristol Robotics Laboratory, University of the West of England, Bristol, United Kingdom; ^3^Department of Biomedical Engineering, Colombian School of Engineering Julio Garavito, Bogotá, Colombia; ^4^School of Engineering, Science and Technology, Universidad del Rosario, Bogotá, Colombia; ^5^Tejido de Sueños, Medellín, Colombia; ^6^Electrical Engineering Department, Federal University of Espirito Santo, Victoria, Brazil; ^7^Faculty of Engineering and Science, Western Norway University of Applied Sciences, Bergen, Norway

**Keywords:** autism spectrum disorder, socially assistive robotics, emotion recognition, participatory design, low-cost social robot

## Abstract

**Introduction:**

Socially Assistive Robotics has emerged as a potential tool for rehabilitating cognitive and developmental disorders in children with autism. Social robots found in the literature are often able to teach critical social skills, such as emotion recognition and physical interaction. Even though there are promising results in clinical studies, there is a lack of guidelines on selecting the appropriate robot and how to design and implement the child-robot interaction.

**Methods:**

This work aims to evaluate the impacts of a social robot designed with three different appearances according to the results of a participatory design (PD) process with the community. A validation study in the emotion recognition task was carried out with 21 children with autism.

**Results:**

Spectrum disorder results showed that robot-like appearances reached a higher percentage of children's attention and that participants performed better when recognizing simple emotions, such as happiness and sadness.

**Discussion:**

This study offers empirical support for continuing research on using SAR to promote social interaction with children with ASD. Further long-term research will help to identify the differences between high and low-functioning children.

## 1. Introduction

Socially Assistive Robotics (SAR) has been receiving considerable attention as an intervention tool to support Autism Spectrum Disorder (ASD) therapies, and innovative healthcare interventions in children with ASD (Cabibihan et al., 2013; Kumazaki et al., 2020). Several strategies using SAR have been developed to improve and promote the development of social skills among children with ASD (Boucenna et al., 2014). Skills such as joint attention (Ramirez-Duque et al., 2018), facial emotion recognition (Yun et al., 2017), verbal and non-verbal communication (Boucenna et al., 2014), and increase self-initiated interactions (Dickstein-Fischer et al., 2018). Even though the evidence for the efficacy of SAR for ASD therapy is promising, there is still not a consensus on how the interactions should be addressed and which robot appearance might be most effective (Feil-Seifer and Matarić, 2009; Costescu et al., 2014; Kumazaki et al., 2020).

A wide range of SAR applications can be found in the literature (Argall and Billard, 2010). However, most of the robots used with ASD populations are off-the-shelf robots, which are not explicitly designed for therapeutic interventions (Vallès-Peris et al., 2018; Randall et al., 2019). Several design techniques have started to be explored, where participatory design (PD) ensures the acceptability and functionality of the robot (Bartneck et al., 2020).

The use of PD methods in technology-based design processes for healthcare allows for highlighting the different experiences and attitudes from different fields. The stakeholders and the target populations are no longer seen as a source to obtain information and requirements to produce results. Instead, they are considered partners with experience with different points of view, which can be a part of the solution (Fletcher-Watson et al., 2018). In this sense, all the actors in the process are recognized as valuable contributors, playing a crucial role in the development of ethical and social considerations. The PD process intention is to achieve products or services that represent the real needs, expectations, and desires of the stakeholders.

PD is particularly promising when transferring knowledge and systems from research to the real-world (Vallès-Peris et al., 2018). Even though PD is inherently reliant on the culture and context of the location in which it takes place, it also represents an opportunity of gathering culture-specific findings and make cross-cultural observations. In this sense, and following our previous work (Ramírez-Duque et al., 2020), this work report the last stage of a PD methodology that aims to present the final guidelines for the design of a social robotic device to be implemented in robot-assisted therapy for children with ASD. Our case study is situated in a Colombian context and the main contributions of this work are: (i) the report of the last stage of a novel 3-year long participatory design strategy, which focuses on designing and assessing multiple physical appearances for a social robot. This methodology is based on well-established generative methods. (ii) An evaluation study of the CASTOR social robot within the Colombian context and Colombian robot-based intervention preferences.

## 2. Background

The implementation of PD has been used in the design of SAR for ASD (Huijnen et al., 2016). For example, surveys about the expectations of the role of SAR in Robot-Assisted therapy for children with ASD are frequently used (Coeckelbergh et al., 2016). These SAR systems are designed to induce tactile interactions that may help to promote social relationships and can be used to mediate interactions between children with ASD and their peers and adults (Simut et al., 2016; Wood et al., 2019). The study by Anzalone et al. (2019) reported three essential aspects during robot design: (i) the social robot's shape should contribute to the reduction of the children's stress during the therapy; (ii) the embodiment of the social robot must allow for physical exploration and interaction with the environment, as well as communication-based on gestures and touch; (iii) social robots in ASD therapy should simplify the internal complexity of social interactions.

Designing a robot for children with ASD should ensure a friendly, playful, and accessible look. The physical structure should be interesting, attractive, and safe during human-robot interactions (Koch et al., 2017). Additionally, since SAR seeks to promote the development of social skills in individuals with ASD, the appearance of the robot and the level of anthropomorphism, or “human likeness” are essential issues (Ricks and Colton, 2010; Scassellati et al., 2012). In consequence, acceptance and perception of the users regarding social robot-based technologies are essential indicators of understanding the effects during the interventions.

As pointed out before to properly integrate social robots in these scenarios, all the stakeholders should be involved (i.e., patients, healthcare professionals, software developers, and caregivers; Bartneck et al., 2020; Ramírez-Duque et al., 2020). By involving the target users during the design process, the acceptance, and effectiveness of the robot could be enhanced (Cho and Ahn, 2016). Therefore, this work is framed within the Compliant Soft Robotics (CASTOR) project, which aims to develop a compliant, soft robot through the use of PD. This is meant to be integrated into the next generation of ASD rehabilitation scenarios based on tangible and affordable SAR. The first stages of the PD implementation were presented in our previous work, which was carried out in four stages: (i) sensitization; (ii) focus group with stakeholders; (iii) generative intervention with children; and (iv) validation and ratification of preliminary findings (Ramírez-Duque et al., 2020). Once the last stage was over, all participants considered that the robot design could be composed of colored lights, different textures, and materials to stimulate the children through other sensory channels. The surveyed population's preferences about the physical features had a predilection for using modular and assembly parts, plastic and textile materials, and a soft body. The participants believed that sound functions, movement of arms, and facial expressions movements (e.g., mouth, eyes, and eyebrow) were essential to improve the child-robot interaction. Additionally, they suggested that the robot could benefit from buttons and screens, different clothes, as well as a face, upper limbs, a microphone, and speakers to allow multimodal communication and interaction with the children (Ramírez-Duque et al., 2020).

Based on these outcomes, this work describes the execution of the fifth and last stage related to the validation of the design process findings to identify the best appearance of a social robot and to assess the acceptability, expectation, and reactions of children with ASD toward a novel SAR tool, known as the CASTOR robot.

## 3. Materials and methods

This work seeks to accomplish two objectives: (i) to identify and gather preliminary information that allows the establishment of the most attractive robot appearance for children with ASD and (ii) to validate the acceptance toward the robot's appearances and functionalities.

In this context, this section is divided into two main parts, the first one describes the last stage of the PD process of CASTOR and the second part describes the experimental protocol for the validation of CASTOR's appearances during emotion imitation and identification tasks.

### 3.1. Part one: Last stage of the participatory design process

In previous works, the authors proposed a PD process entailing the fourth stages (i.e., sensitization, focus groups, generative interventions, and preliminary validation). This work addresses the last stage to identify the appearances that the social robot should have, as well as their validation in a clinical scenario. The design criteria and the methodology applied to achieve the CASTOR's appearances are described below.

#### 3.1.1. Design criteria

In order to be consistent with the first four stages of the PD process, this work was developed under the same design criteria that were previously used (Ramírez-Duque et al., 2020). Overall, the PD process's central premise was the active participation and involvement of the different stakeholders. In this sense, an immersive experience at the clinic allowed the definition of the physical appearances that the robot should have. In particular, caregivers, therapists, and children provided their insights and opinions on this.

In this scenario, this work maintains the design premises founded in our previous work (Ramírez-Duque et al., 2020). First, the robot must provide a safe, enjoyable, and non-judgemental environment. Second, it must allow for simple social interaction, as well as comfortable interaction with the child. Thirdly, the robot must not be expensive, and it must be resistant to allowing free interaction with the child. The robot must be able to be controlled and monitored remotely to avoid interfering with therapy with the children. And finally, the physical appearance of the robot must be able to be easily cleaned and exchanged.

#### 3.1.2. CASTOR's appearances design

The CASTOR's appearance design was based on inclusive and participatory design techniques. This involved all the expectations, sensations, perceptions and reactions of children with ASD (between 3 and 9 years old), their caregivers, and the CASTOR team. The CASTOR team includes the creative enterprize specializing in inclusive design “*Tejido de Sueños,”* a group from the Howard Gardner Clinic, comprising healthcare and administrative specialists, and finally, an engineering group from the University “*Escuela Colombiana de Ingenier*í*a Julio Garavito*.”

**Figure 1:**
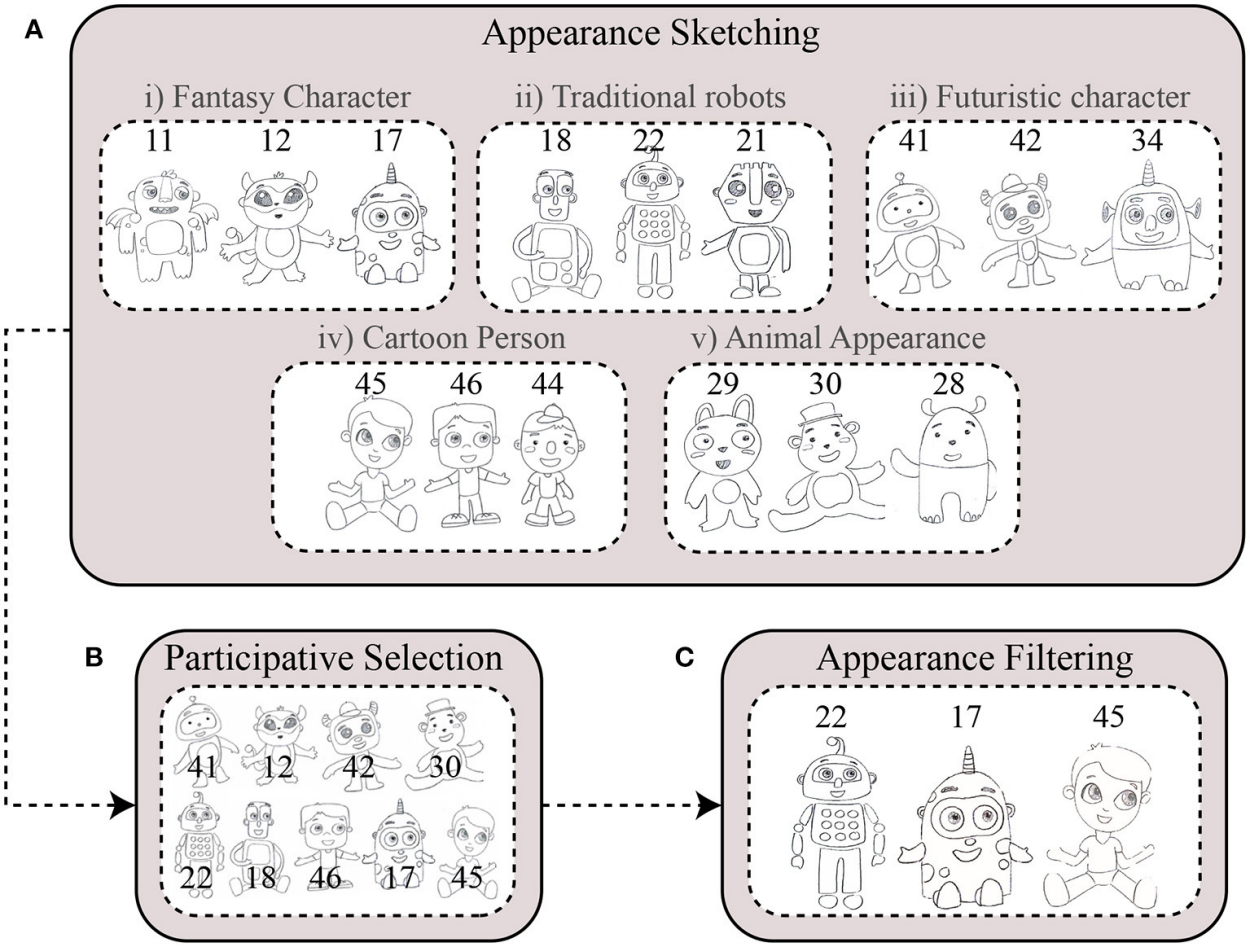
Selection process of the CASTOR's appearance. The initial set of robot appearances consisted of 50 sketches that were selected and filtered through a participatory process with stakeholders. **(A)** Appearance sketching. **(B)** Participative selection. **(C)** Appearance filtering.

As a first step, the enterprize “*Tejido de Sueños”* designed 50 sketches considering the guidelines provided by the interventions with children and stakeholders. The initial set of sketches aimed to provide multiple ideas for the appearances of the robot. The sketches were divided into five categories: (i) cartoon persons; (ii) traditional robots; (iii) futuristic characters; (iv) animal-like appearance; and (v) monsters/fantasy characters. As the second step, to validate the appearance ideas with the stakeholders, two stages were carried out: (i) appearance assessment, and (ii) participatory selection (see Figure 1).

**i. Appearance assessment**. This phase aimed to identify which sketches were the more engaging ones. An evaluation matrix with six criteria was proposed (see Table 1). The criteria were designed from the requirements identified in the previous stages at the clinic. Fifty-two volunteers, including caregivers, parents, and interested parties, participated in this process. Children with ASD were not included in this first step because the activity might be difficult for them. Each participant was instructed to give a score from 1 to 5 for each requirement in the evaluation matrix. For this, each participant received all the sketches listed from 1 to 50 (see Figure 1A), and the scores for each illustration were obtained through a simple average between all the requirements. From the outcomes, nine sketches with the highest score and interest were selected and used for the next stage (see Figure 1B).

**Table 1:** Sketches evaluation matrix.

**Appearances requirements**	**Sketch #1**	**Sketch #2**	**...**.	**Sketch #50**
Friendly, peaceful, and empathetic appearance				
Facilitates the recognition of facial expressions and emotions				
Encourages eye contact				
Attractive design for children				
Ability to customize with accessories to determine gender or personality				
Encourages physical contact through forms or materials				
Facilitates imitation and interaction				

**ii. Participatory selection**. The participatory selection phase involved the children in the appearance selection process. This phase sought to obtain the three most notable appearances (see Figure 1C). With this idea, simple generative activities were designed. Nineteen children with ASD participated.

This phase was composed of two activities with a set of nine cards of the same size, which were prepared with the nine sketches already chosen in the previous stage. The first activity consisted of sorting the cards placed on a table in front of the children. After providing a short time to look at the different robots, a therapist asked them to take the card that they liked the most. Once they picked a sketcher, the corresponding card was removed from the table, and the action was repeated successively until a card ranking was made. The second activity consisted of matching adjectives to the cards. The cards were placed again in front of the children, and another set of cards depicting different adjectives was presented. This set was composed of six cards representing several adjectives through emotions and pictographs. The used adjectives were: *cute, ugly, hero, villain, friendly, and fearful*. The therapist invited the children to match each sketch with an adjective, looking for any association or feeling they could have for each appearance.

Finally, the appearance assessment and the participatory selection phases resulted in three sketches. In this context, the second objective of this study sought to validate these appearances and the CASTOR's functionalities. Specifically, the appearances were assessed regarding their ability to elicit learning to express emotions through recognizing and imitating tasks. This study also focused on validating the children's acceptance of the CASTOR's design and appearance.

### 3.2. Part 2: Experimental protocol for the CASTOR's validation

This section describes the experimental protocol designed to address the proposed objectives. The validation study was also conducted at the Howard Gardner clinic, where CASTOR was deployed with three different appearances. For this, three groups were defined to evaluate each of the appearances separately: *Fantastic group, Robot Group*, and *Human group*. The following sections describe the ethics statement, the participants that were allowed to participate in the study, the experimental design, and the experimental procedure.

#### 3.2.1. Ethics statement

The Colombian School of Engineering Julio Garavito's ethics committee approved the protocol. The children's parents were informed about the scope and purpose of the experiment, and written consent was obtained from each of them before the study. The children's counselor was consulted and informed about the activities to be performed and gave suggestions for the improvement of the protocol.

#### 3.2.2. Participants

A total of 21 children diagnosed with ASD were enrolled in this study, forming three different groups each one of seven participants: a fantastic group (F, one female, six males, 8.57 ± 3.01 years old); a robot group (R, two females, five males, 7.28 ± 2.81 years old); and a human group (H, two females, five males, 7.83 ± 1.95 years old). All children were randomly assigned to the experimental groups. The participants were selected according to the inclusion and exclusion criteria described below:

**Inclusion Criteria:** Children with ASD between 5 and 10 years old. Children who obtained consent were informed by their legal representative.

**Exclusion Criteria:** Children that exhibited any visual, auditory, or cognitive impairment that impeded the correct understanding of the activity were excluded. Additionally, children who present any comorbidities such as Fragile X Syndrome or Down Syndrome were not able to participate in the study.

Once the children's parents agreed that their child was going to participate in the study, each child was randomly assigned to one group. The protocol supervisor explained the conditions under which the experiment was going to be performed. All participants were free to abandon the study whenever they decided to do it. The children did not know the other experimental conditions (i.e., the children of the fantastic group did not know the different appearances of the CASTOR and had no contact with them).

#### 3.2.3. Experimental procedure

This study was based on emotion imitation and recognition tasks to assess the ability of CASTOR to facilitate emotion learning in children. A control phase (i.e., without robot) and an intervention phase (i.e., with robot) were performed for each robot's appearance to identify the effects of CASTOR and its appearances during the tasks. In both control and intervention phases, the children only had three attempts to perform each activity (i.e., imitation and recognition). If the children succeeded in the task on the first attempt, they received three points. If the children required more than one attempt, one unit score decreased until all three attempts were completed. Otherwise, no points were summed up to their score. It is essential to highlight three aspects: (i) the children did not know about the scoring system; (ii) no child had seen the robot before; and (iii) the CASTOR's appearance did not change within the same group.

**Figure 2:**
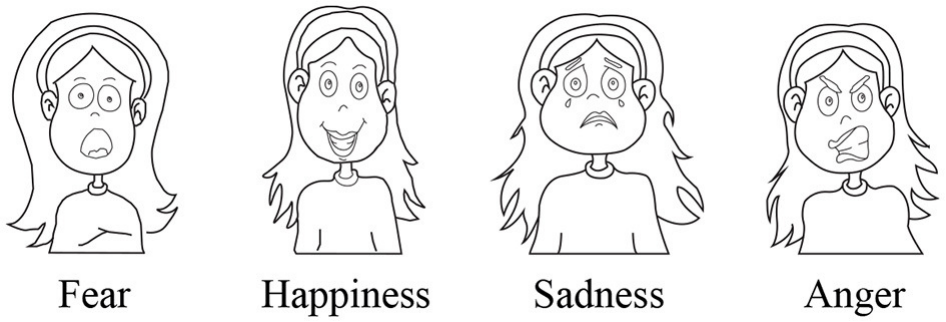
Basic emotion cards used in the first phase of the study. A therapist asked the participants to identify the emotions displayed in the cards.

In the control phase, the sessions were conducted only with the therapist. First, the children were instructed to identify four raw emotions (i.e., happiness, sadness, anger, and fear) using four cards (see Figure 2). Then, the children were asked to imitate the emotions shown on the cards.

The intervention phase was divided into two activities: familiarization and intervention. The familiarization phase was carried out considering that children with ASD might have difficulty accepting changes to their environment and their daily routine (Hill and Frith, 2003). Therefore, this phase allowed us to introduce and socialize the robot with the children and thus integrate it into their environment. The children were able to freely explore the robot aiming to provide safety and confidence to the children. At this stage, CASTOR asked the children what their name was to establish a relationship between them.

**Figure 3:**
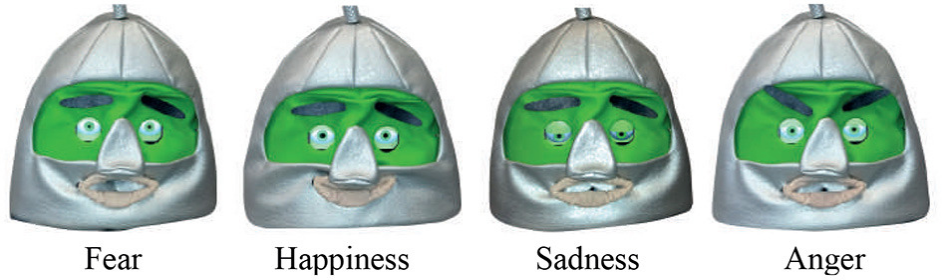
CASTOR's facial emotional expressions. The robot was introduced during the third phase, where a therapist asked the children to identify and imitate the robot's gestures/emotions.

Afterwards, the intervention was executed with an average duration of 12 min. In this phase, CASTOR accompanied the therapist in the tasks. The cards were no longer required. The robot began performing the previously mentioned four raw emotions (see Figure 3), and the therapist asked the children to identify them. Once the four emotions were completed, the robot started to perform one of the four emotions again. At this point, the therapist asked the children to imitate the emotion being carried out by the robot.

#### 3.2.4. Experimental setup

**Figure 4:**
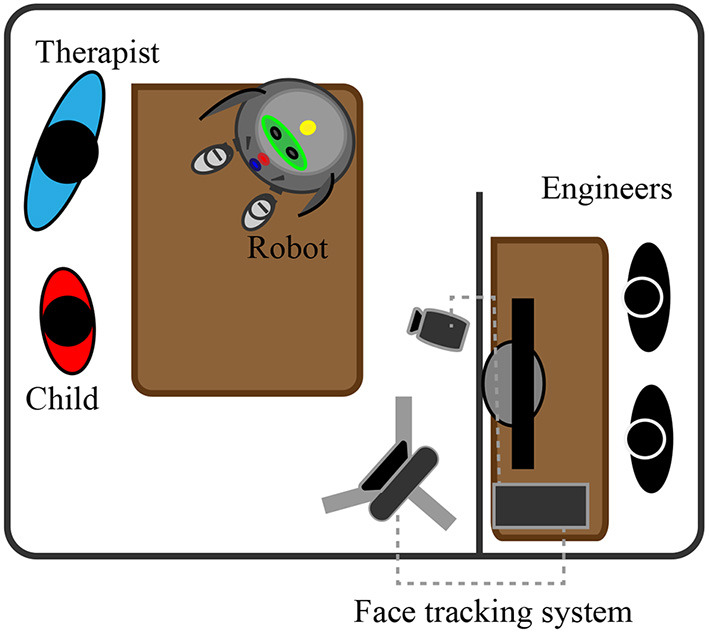
Experimental environment at the rehabilitation center. A face tracking system allowed the quantification of children's behaviors. A group of engineers remotely controlled CASTOR from a hidden area.

The study took place at the Howard Gardner Comprehensive Rehabilitation Clinic in an adapted room. The room had two divisions an experimental area and a remote control area. The standard layout can be seen in Figure 4. The cameras used to record the sessions had wide-angled lenses to ensure that the child was always in the field of view. The facial expressions, eye gaze, and children's movements were captured during the experiments. In the hidden control room, the researchers controlled the robot's movements through a chat-bot interface designed with the telegram bot API.

During control sessions, the robot was hidden, and the raw emotion cards were placed on the table. For the familiarization and intervention phases, the robot was placed on the table.

#### 3.2.5. Variables

The experimental protocol contemplates the quantitative measurements related to the variables that can be recorded and stored with the information provided by the therapist and the system. This information indicates the performance of the child in the session. It is worth mentioning that both attention and performance in imitation/recognition tasks are commonly used variables to assess robotic tools for ASD therapy (Robins et al., 2006; van Straten et al., 2018). Furthermore, interviews were not used as the children came from a very diverse group, and this kind of measurement often requires high-functioning individuals (Kumazaki et al., 2017).

##### 3.2.5.1. Variables measured by the face-tracking system

The face-tracking system implements an architecture for distributed video acquisition and processing. Two RGB-Depth sensors (Kinect 2, Microsoft) were used to acquire information from the child and the robot. This data is then used to extract nonverbal cues from the child's face, such as head and body motion, head pose, eye gaze, visual contact, and visual focus. To this end, a processing pipeline entailing multiple modules was used: (1) two Convolutional Neural Networks for face detection and recognition, and (2) a Conditional Local Neural Fields statistical model for head pose and eye gaze estimation (Ramírez-Duque et al., 2019). Overall, two metrics were obtained from this system. (1) *Visual contact* measures the time during which the patient makes eye contact with the therapist. (2) *Device Attention* measures the time during the session in which the patient looked at the robotic device.

##### 3.2.5.2. Variables recorded by the therapist

The therapist recorded the number of times the child correctly identified the emotions shown in the cards or the robotic mediator, as well as the number of times the child imitated those emotions.

#### 3.2.6. Statistical analysis

The software package SPSS (IBM-SPSS Inc., Armonk, NY, USA) was used for the statistical analysis.

Considering the small sample size, non-parametric statistics (i.e., Kruskal Wallis test and Wilcoxon signed-rank test) were carried out to analyze the effects of the appearance in the performance and attention between control and intervention phases. In the same way, the Friedman test and the Bonferroni *post-hoc* test were performed to determine the existence of significant differences among the emotions.

## 4. Results

This section describes the results obtained during the participatory design (PD) process, as well as the results of the validation study with 21 children with ASD.

### 4.1. Participatory design

**Figure 5:**
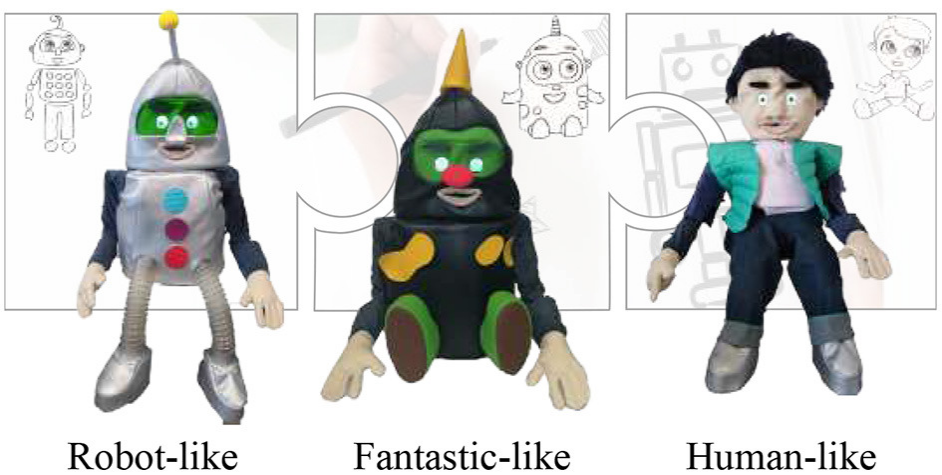
CASTOR's appearances obtained from the participatory design process with autism community at the Howard Gardner Clinic.

The PD outcomes from the CASTOR appearances are presented in Figure 5. Three appearances denominated as human-like, fantastic-like, and robot-like were chosen. These appearances were rendered in the social robot using imitation leather.

### 4.2. Emotions recognition and imitation

Table 2 summarizes the average performance score for each group of participants (i.e., control and intervention), each appearance type (i.e., fantastic, robot, and human like), and each emotion (i.e., happiness, sadness, anger, and fear). These values were estimated by computing the sum of the scores obtained throughout the proposed activities for each assessed emotion, i.e., identification and imitation. In the same way, Table 3 shows the obtained statistics for the Kruskal-Wallis tests. These tests compared the performance scores under the same group and activity type (i.e., identification and imitation) among all appearances. Table 3 describes obtained *p*-values, H-scores, and degrees of freedom. In particular, no statistically significant differences were found, and thus the effect size is not reported.

**Table 2:** Performance scores (mean ± std) of children under emotion identification and imitation task.

**Groups**		**Happiness**	**Sadness**	**Anger**	**Fear**
**Identification**					
**Identification task in control and intervention phases for the**					
**three groups**					
	Fantastic	3.00 ± 0.00	2.50 ± 1.22	3.00 ± 0.00	2.20 ± 0.85
C	Robot	2.57 ± 0.78	2.71 ± 0.75	3.00 ± 0.00	2.57 ± 0.78
	Human	2.71 ± 0.48	3.00 ± 0.00	2.28 ± 1.25	1.43 ± 1.27
	Fantastic	3.00 ± 0.00	2.33 ± 1.21	2.00 ± 1.51	1.66 ± 1.51
I	Robot	2.71 ± 0.75	2.71 ± 0.48	2.14 ± 1.21	2.14 ± 1.46
	Human	2.57 ± 1.13	2.57 ± 1.13	2.14 ± 1.46	1.85 ± 1.46
	Average	2.76 ± 0.53	2.59 ± 0.93	2.07 ± 1.27	2.11 ± 1.31
**Imitation**					
**Imitation task in control and intervention phases for the**					
**three groups**					
	Fantastic	3.00 ± 0.00	2.33 ± 1.21	2.83 ± 0.41	3.00 ± 0.00
C	Robot	2.71 ± 0.75	2.57 ± 1.13	2.71 ± 0.75	2.14 ± 1.35
	Human	2.71 ± 0.75	2.14 ± 1.46	2.71 ± 0.75	2.57 ± 1.13
	Fantastic	2.14 ± 1.46	2.00 ± 1.54	2.00 ± 1.54	1.85 ± 1.60
I	Robot	2.42 ± 0.75	2.42 ± 0.58	1.71 ± 1.60	2.57 ± 1.13
	Human	2.42 ± 1.13	2.14 ± 1.46	2.85 ± 0.38	1.28 ± 1.46
	Average	2.57 ± 0.94	2.23 ± 1.24	1.97 ± 1.42	2.54 ± 0.96

C, Control; I, Intervention.

**Table 3:** Kruskal-Wallis test results for appearances comparison under the same group (i.e., intervention group and control group) and activity (i.e., Identification and imitation).

**Variable**	***p*-value**	**H-score (df)**
Control group Identification task	0.898	0.021 (2)
Intervention group Identification task	0.859	0.304 (2)
Control group Imitation task	0.993	0.013 (2)
Intervention group Imitation task	0.651	0.857 (2)

The *p*-value, H-score, and the degrees of freedom (df) are reported. ^*^Denotes significant differences.

Similarly, Table 4 shows the obtained statistics for the Wilcoxon tests. Specifically, these tests compared: (i) all emotions between activities under the same group and (ii) all emotions between groups under the same task. Table 4 describes the obtained *p*-values, Z-scores, and degrees of freedom.

**Table 4:** Wilcoxon test results for all emotions comparing against activities and groups.

**Variable**	***p*-value**	**Z-score (df)**
Identification vs. imitation for control group	0.952	−0.061 (83)
Identification vs. imitation for intervention group	0.276	−1.091 (83)
Intervention vs. control group for identification task	0.003^**^	−2.216 (83)
Intervention vs. control group for Imitation task	0.004^**^	0.857 (83)

The *p*-value, Z-score, and the degrees of freedom (df) are reported.

*** *p* < 0.001;

** *p* < 0.01;

* *p* < 0.05.

Given the previous results, the scores for the same emotion type were then analyzed together, regardless of the group or activity type. Thus, Friedman test was carried out to compare the total scores among the four emotions. The results showed significant differences between the emotions at *p* < 0.05 (*p*-value = 0.003, χ^2^ = 13.429, *df* = 3). In this case, the Kendall's W value was calculated to obtain the effect size. In particular, a value of 0.21 was obtained and according to Cohen's interpretation, it represents a small effect. Furthermore, Bonferroni *post-hoc* tests were carried out, finding significant differences almost against all the emotions, except for Fear vs. Anger. The obtained *p*-values are reported in Table 5.

**Table 5:** *Post-hoc* comparisons of emotions using the Bonferroni *post-hoc* test.

**Happy**	**Sadness**	**Fear**	**Anger**	
Happiness	–	0.004^**^	0.012^*^	0.022^*^
Sadness	0.004^**^	–	0.019^*^	0.024^*^
Fear	0.012^*^	0.019^*^	–	0.072
Anger	0.022^*^	0.024^*^	0.072	–

*** *p* < 0.001;

** *p* < 0.01;

* *p* < 0.05.

**Figure 6:**
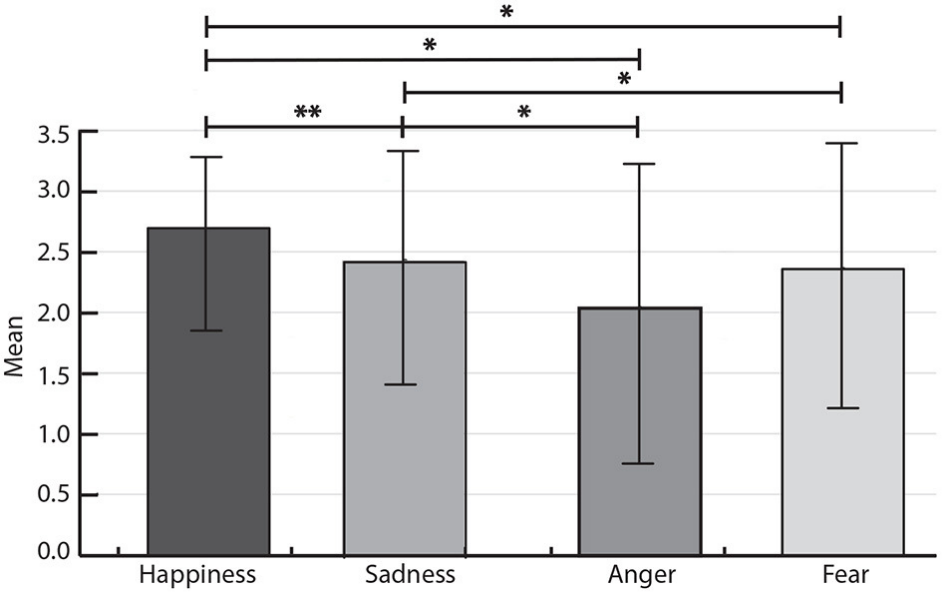
Comparison of participants' average score between emotion, regardless of the group of the emotional gestures on the CASTOR robot (i.e., imitation and identification). ^***^p < 0.001; ^**^p < 0.01; ^*^p < 0.05.

Figure 6 reported the differences between the score during imitation and identification of the fourth emotion on the social robot CASTOR. The significant differences between emotions were indicated with the asterisk.

### 4.3. Child's attention assessment

The children's attention was estimated to determine the most attractive CASTOR's appearance and the acceptability of the robot's presence during the therapy. Specifically, a face tracking system calculated the children's eye gaze offline. During control trials, the children's attention to the therapist was extracted, and during intervention trials, both the attention to the robot and the therapist were estimated.

**Figure 7:**
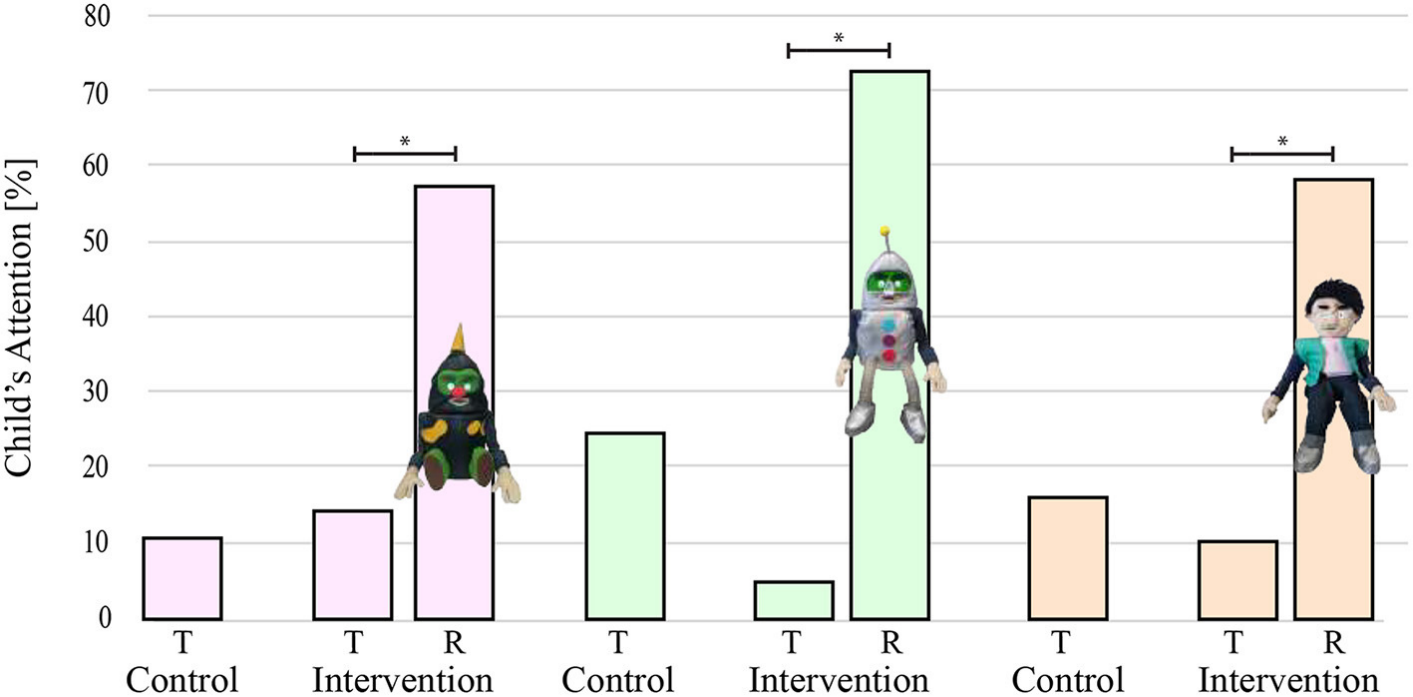
Mean values of children percentage of attention for the three CASTOR appearances. T stands for therapist attention and R stands for CASTOR attention. Asterisks indicate significant differences.

Figure 7 illustrates the mean percentage of children's attention during the session over the three appearance groups for both trial types, i.e., control and intervention.

## 5. Discussion

One of the key goals of this work was to prioritize the users' wellbeing and experience rather than focusing on the technological development. Therefore, the implemented activities supposed an opportunity to spend time with the community and understand the atmosphere (e.g., healthcare institution, therapies facilities), the issues with conventional treatment, the concerns and the suggestions of all stakeholders. Through the implementation of the last stage of the participatory design process, a relationship between trust and understanding was established between the children with ASD, the parents, the clinicians and the researchers. The users' acceptance and perception were the most important aspects, and thus they were maximized during the design and development of the CASTOR's appearances.

### 5.1. Participatory design

The data generated during the last phase of the participatory process (PD) were analyzed. For this, we observed video recordings allowing the team to understand the activity's atmosphere and identify the main aspects of the CASTOR's appearances design. The PD outcomes revealed that children with ASD- in our sample- preferred robots that looked like fantastic characters, with a preference for a neotenous appearance and exaggerated features. Additionally, preference was expressed for a robot that had an appearance between a cartoon and a fantastic animal. In the same way, the children appreciated the sketches that looked like a child. Hence, three human-like, fantastic-like, and robot-like appearances were identified.

On the other hand, it was considered that the social robot would be used in a clinical environment for the CASTOR appearance design and development. Therefore, the appearance reconstruction was designed to guarantee easy cleaning and quick replacement. In this context, these appearances were rendered in the real robot using imitation leather.

Concerning the role of the social robot, the PD process also revealed that the stakeholders (i.e., caregivers and therapists) imagined the robot as a mediator and facilitator (i.e., as a natural extension of the physical resources in the intervention). In this sense, the parents and therapists identified that (i) verbal communication, (ii) learning to express emotions and feelings, (iii) encouraging eye contact, and (iv) self-care activities were the primary skills that can be stimulated in robot-assisted therapies. Besides, participants suggested that CASTOR should interpret the child's thinking, emotions, and intentions. Notwithstanding, the participants also reported that they feared that the robot could generate stress, fear, or frustration in the children.

### 5.2. Emotions recognition

According to the PD outcomes, an evaluation study was conducted to find the most attractive appearance for the children with ASD and to make the social robot's first approach to a real clinical environment. The study involved emotion recognition and imitation aiming to validate the acceptability of the social robot and its integration into the activities with the clinicians. In this evaluation, three groups for each appearance with children with ASD were enrolled. During this task the Kruskal Wallis test results showed that there were no statistically significant differences between appearances (i.e., robot, fantastic, and human). In this context, the three groups were considered homogeneous, and it was concluded that the CASTOR's appearances did not influence the children's performance. The above was expected given the appearance design process that was done through the PD.

Regarding the children's performance between the two activities (i.e., identification and imitation), no statistically significant differences were found between the control and intervention groups, as is shown in Table 4. This means that the children's performance is not changed or improved by the robot. These results are consistent with the study by Yun et al. (2017), which reported that facial emotion recognition was not significantly different between the robot and the control groups. On the other hand, the study by So et al. (2018) reported that it is not clear whether the robot was better than humans (e.g., peers or therapeutics) at administering the assessments and training gestures with children with ASD. Moreover, although there are no significant differences in performance, the study by Zorcec et al. (2018) reported that after eight sessions, parents stated that recognition and appropriate reaction to happy and sad emotions was used in everyday life. With the above, it would be possible to state that CASTOR can be a therapy aid and help as an assistive tool in traditional methods.

Participants displayed difficulty identifying and generalizing certain emotional expressions. Statistically significant differences were found between emotions, as illustrated in Figure 6. In general, happiness and sadness were correctly labeled and matched most consistently. Although anger and fear were frequently labeled correctly, participants often confused anger expression with fear. Consequently, no statistically significant differences in the *post-hoc* test were found between fear and anger. These findings coincide with similar studies that explore the facial expressions of social robots (Sosnowski et al., 2006; Saldien et al., 2010; Salvador et al., 2015), where they reported that complex emotions (e.g., fear and anger) were found more challenging to identify and discriminate. Another study without using social robots reported that these emotions are not similar for neurotypical individuals, and it is easier to identify (Marsh et al., 2005). Future studies should focus on identifying the possible reasons that may cause these differences.

These results indicate two main things (i) the CASTOR's anger expression needs to be improved, and (ii) another study with this improvement is required to corroborate these findings. Regarding CASTOR's capability to portray facial emotions, there are only subtle differences between expressions of fear (e.g., pupil dilation, mouth widening) and anger (e.g., pupil contraction). Children can overlook these discrepancies, generating confusion among the emotions, as evidenced in the *post-hoc* test results. However, therapists reported that these subtle facial details make CASTOR's expressions unique and can be used to highlight differences between emotions when teaching emotional recognition skills to children with ASD. Specifically, eye contact can be trained to improve the children's identification of the four raw emotions.

### 5.3. Child's attention assessment

The results presented in Figure 7 showed that the robot-like outfit presented 72.56% of the child's attention in the intervention phase corresponding to the maximum children's percentage of attention during all sessions and activities. The above suggests that the CASTOR's appearance, with the larger attention and acceptance, was the robot-like one. Likewise, previous studies have shown that children with ASD prefer robot-like appearances rather than highly human-like appearances (Kumazaki et al., 2017). Therefore, for future user studies, using the CASTOR's robot-like appearance should be considered and recommendable.

The results indicated that the therapist's attention considerably decreased between control and intervention trials. The children's attention was more notable and constant in the intervention phase. In fact, the children's attention increased by around 50% during the session assisted by the therapist and CASTOR. Statistically significant differences were found for each activity (e.g., imitation and recognition). This indicates that CASTOR's appearance impacts the children's attention with the robot's presence, e.g., children devote more attention to the activities when interacting with the robot. Also, even only with the CASTOR's arrival, the attention was greater. These suggest that the child improved their attention, eye contact, and interest in the therapy with the CASTOR's presence.

On the other hand, it was observed that for each of CASTOR's appearance, a minimum percentage of attention to the therapist was maintained. These results are consistent with Srinivasan et al. (2016) and Ramirez-Duque et al. (2018), who reported that the children devoted maximum attention to the robot rather than the therapist. Besides, they reported that the children continued to devote the most attention to the robot throughout the treatment sessions without losing interest. These findings should be considered when designing a long-term therapy with CASTOR. Although the children may be more interested and comfortable in therapy, the visual fixation on the robot may affect the child's opportunities to engage with social patterns.

Finally, it is essential to note that at the end of the sessions, the therapists stated that the children felt safe, calm, comfortable, and interested in the company of the robot within the therapy. They even reported that some children with the presence of the CASTOR reduced their anxiety levels. This fact was observed and reiterated by the researchers, peers, and caregivers through the recordings. With the above, it is possible to determine a positive acceptance of the child toward CASTOR regardless of its appearance. This shows the importance of participatory design, which allowed us to see rewarding results. Similarly, it could be noted that the social robot CASTOR was well integrated into the activities with the clinicians and was greatly accepted by the children.

## 6. Conclusions

This article presented a study in a clinical setting using a novel social robot with three different appearances (e.g., human-like, fantasy-like, and robot-like) implemented and designed through an inclusive and participatory design (PD) process. Implementing PD is not just a methodology to improve and enhance a product's final design but also an opportunity to understand and gain knowledge about the community's context and to build trust and confidence between researchers and the community. The current state-of-the-art reveals some constraints regarding fragile structures and high acquisition costs of existing social robots. On the other hand, CASTOR takes on additional significance regarding the development of robotic systems in Latin American countries. In particular, the community's awareness of technology and robotics adoption in healthcare is lower than in countries like the USA and Japan.

The main objective of this work was to identify the most attractive CASTOR's appearance and determine the CASTOR's functionality and acceptability in ASD therapies. Thus, this study presented the results from one emotion recognition task with two variations, i.e., identification and imitation. These variations were relevant for this study due to the importance of emotion recognition to establish relationships with others and the fact that it plays a critical role in everyday communication. Specifically, therapists asked the participants to identify four raw emotions (e.g., happiness, sadness, anger, and fear) using images or a social robot.

Regarding the study results, the participating children were confused when recognizing complex emotions (e.g., fear and anger). In contrast, with simple emotions (i.e., sadness and happiness), they made an outstanding performance. These emotions were correctly labeled and matched. Results also showed that the children looked at CASTOR more than the therapist. However, the children kept most of their attention on the therapist in both control and intervention trials, mainly due to adult-seeking and acceptance-searching behaviors. Moreover, the therapists played an essential role during sessions, as they helped to build the relationship and trust between the children and the robot. In other words, the active participation of therapists and the relationship between the children, the therapist, and the robot are essential to ensure the intervention's success.

In terms of appearances, the one that attracted the most attention was the robot-like appearance. However, the rationale behind this is yet to be explored with further studies. A hypothesis around this topic states that children prefer robots with more aesthetically pleasing characters or more authoritative figures. On the other side, it is essential to point out that no matter the CASTOR's appearance, the child felt safe, calm, and comfortable during the CASTOR's presence. Also, some children reduced their anxiety levels.

On the other hand, the CASTOR design guarantees an assistive robot with simplified and realistic features that allow simple social interaction and more comfortable interaction with children with ASD. This robot is an aid for teaching emotion recognition and imitation, where children interact physically and cognitively with the robot on their terms. In this way, CASTOR serves as a social mediator, engaging children with autism in verbal and non-verbal communication scenarios with another person (e.g., parents, caregivers, or playmates). These results support the idea that robots are active reinforcement agents in semi-structured behavior for children with ASD.

This study offers empirical support for continuing research on using SAR to promote social interaction with children with ASD. Further long-term research will help to identify the differences between high and low-functioning children. Moreover, future work will address the implementation of a physical interaction study to gather tactile information between children and CASTOR using the robot-like appearance. Likewise, CASTOR functionalities will benefit from more complex behaviors, such as body motion and proprioceptive awareness. Also, it would be interesting to test the relative improvements gained from a robot-assisted intervention compared to more traditional interventions that do not include robots, adding a control group to the procedure.

## Data availability statement

The original contributions presented in the study are included in the article/supplementary material, further inquiries can be directed to the corresponding author/s.

## Ethics statement

The studies involving human participants were reviewed and approved by the Colombian School of Engineering Julio Garavito's Ethics Committee approved the protocol. The children's parents were informed about the scope and purpose of the experiment, and written consent was obtained from each of them before the study. The children's counselor was consulted and informed about the activities and suggested improving the protocol. Written informed consent to participate in this study was provided by the participants' legal guardian/next of kin.

## Author contributions

MP-B and SS did material preparation, data collection, and analysis. The first draft of the manuscript was written by MP-B and SS, and all authors commented on previous versions of the manuscript. All authors contributed to the study conception and design, read, and approved the final manuscript.
